# Prognostic factors for the development of systemic lupus erythematosus in patients with immune thrombocytopenia

**DOI:** 10.1186/s13075-022-02901-y

**Published:** 2022-09-06

**Authors:** Soo Min Ahn, Eun-Ji Choi, Ji Seon Oh, Yong-Gil Kim, Chang-Keun Lee, Bin Yoo, Seokchan Hong

**Affiliations:** 1 Department of Rheumatology, Asan Medical Center, University of Ulsan College of Medicine, Seoul, Republic of Korea; 2 Department of Hematology, Asan Medical Center, University of Ulsan College of Medicine, Seoul, Republic of Korea; 3 Department of Information Medicine, Big Data Research Center, Asan Medical Center, Seoul, Republic of Korea

**Keywords:** Thrombocytopenia, Immune thrombocytopenia, Systemic lupus erythematosus, Antinuclear antibody, Organ bleeding

## Abstract

**Background:**

Patients with immune thrombocytopenia (ITP) have a risk of developing systemic lupus erythematosus (SLE). We sought to examine the clinical characteristics of patients with primary ITP who later developed SLE and identified the risk factors for the development of SLE.

**Methods:**

We retrospectively examined patients who were diagnosed with primary ITP at a tertiary hospital between August 2001 and November 2019. We compared the clinical characteristics according to the development of SLE. Logistic regression analysis was performed to identify the factors associated with the development of SLE.

**Results:**

Of 130 patients with primary ITP, 10 (7.7%) were later diagnosed with SLE during follow-up (median, 30 months [IQR, 15.5–105]). The presence of skin bleeding, organ bleeding, lymphocytopenia, anemia, and antinuclear antibody (ANA) positivity (≥ 1:160) were more common among patients who later developed SLE than did those who did not develop SLE. Multivariate analysis showed that young age (< 40 years; odds ratio [OR], 6.307 [95% confidence interval (CI), 1.114–34.908]; *P* = 0.035), organ bleeding (OR, 13.672 [95% CI, 2.437–76.689];* P* = 0.003), and ANA positivity (1:160; OR, 6.638 [95% CI, 1.399–31.504]; *P* = 0.017) were significantly associated with the development of SLE.

**Conclusions:**

Young age (< 40 years), organ bleeding, and ANA positivity (≥ 1:160) were risk factors for the development of SLE in patients with primary ITP. Close follow-up is needed to detect the development of SLE in patients with ITP and the abovementioned risk factors.

## Background

Immune thrombocytopenia (ITP) is an acquired autoimmune disease characterized by isolated thrombocytopenia and normal or increased numbers of bone marrow megakaryocytes. ITP is classified as primary or secondary according to the presence of underlying etiology. Secondary ITP is related to various conditions that can influence the development of thrombocytopenia, and includes autoimmune diseases, immunodeficiency, drugs, or infection [1].

Systemic lupus erythematosus (SLE) is a chronic systemic autoimmune disease with variable clinical features, and it can be one of the causes of secondary ITP [2]. The prevalence of ITP has been reported to be between 7 and 30% in patients with SLE [3–5]. The pathogenesis of ITP and the exact mechanism of immune-mediated thrombocytopenia in SLE remains unknown; however, several studies reported that ITP and SLE share a common genetic predisposition [6–9], and other studies suggested that some patients presented with ITP as the initial manifestation of SLE [10, 11]. Furthermore, a recent population-based cohort study reported that patients with idiopathic ITP had a 26 times higher risk for the development of SLE than the control population [12].

Because SLE involves various organs and causes heterogeneous clinical symptoms, the diagnosis of SLE is often difficult, especially in individuals who have subtle symptoms (e.g., rash, synovitis) in the early stages [6]. Yet, because significant predictive risk factors related to the future development of SLE in patients with primary ITP are not known, identifying the clinical features associated with the occurrence of SLE during the disease course of ITP would be important. In this study, we examined the clinical characteristics of patients with primary ITP who later developed SLE and identified the risk factors for the development of SLE in primary ITP.

## Methods

### Study population and definitions

In this retrospective study, we reviewed the data of patients who were newly diagnosed with primary ITP between August 2001 and November 2019 at Asan Medical Center, a tertiary referral hospital in Seoul, South Korea. Primary ITP was defined as a platelet count of less than 100 × 10^9^/L in the absence of other possible causes of thrombocytopenia [13]. Accordingly, patients who had other causes or disorders that may be associated with thrombocytopenia were excluded. All patients underwent bone marrow examination and test for antinuclear antibody (ANA) at the time of ITP diagnosis. Of them, patients who were followed up for at least 1 year after the diagnosis of primary ITP were included in the study. The total study period, including the follow-up period for these patients, was from August 2001 to March 2021.

The following data were collected from the electronic medical records: demographic information (sex, age, body mass index, comorbid diseases [hypertension, diabetes mellitus]), bleeding-related clinical symptoms (skin, mucosa, or organs) [14], baseline laboratory data, and bone marrow examination results. In addition, medications and treatment responses from ITP diagnosis to one year after diagnosis were also investigated. Treatment responses was classified into “complete response,” “partial response,” and “no response” according to the definition of the international working group in 2009 [13].

SLE was diagnosed according to the revised 1997 American College of Rheumatology classification criteria [15]. To exclude patients with thrombocytopenia due to SLE, patients who were diagnosed with SLE within 1 year after the ITP diagnosis were excluded. Disease activity was measured by the SLE disease activity index 2000 (SLEDAI-2K) at the time of SLE diagnosis [16].

### Statistical analysis

Chi-squared test and Fisher’s exact tests were used to compare categorical data. Continuous values are expressed as mean ± standard deviation or as median (interquartile range [IQR]) and were compared using Student’s *t*-test for parametric data and Mann–Whitney *U* test for nonparametric data. To identify the risk factors for the development of SLE, univariate and multivariate logistic regression analyses were performed and the results are reported as odds ratios (ORs) and 95% confidence intervals (CIs). Due to the rarity of SLE events, we estimated ORs using penalized maximum likelihood estimation to minimize bias in the multivariate model. Variable that had a *P*-value of < 0.1 on univariate analysis were selected for multivariate analysis, and a stepwise backward elimination procedure was used. Statistical significance was set at *P*-value < 0.05. All statistical analyses were performed in IBM SPSS Statistics for Windows, version 21.0 (IBM Corp., Armonk, NY, USA).

## Results

### Study population

A total of 130 patients with primary ITP were included in this study. The clinical characteristics of the patients at the time of ITP diagnosis are shown in Table 1. The median age was 52 years (IQR, 34–61), and 90 (69.2%) patients were female. The mean platelet count at the diagnosis of ITP was 13 × 10^9^/L, and severe thrombocytopenia (< 20 × 10^9^/L) was found in 84 (64.6%) patients. Of the total patients, 10 (7.7%) patients were eventually diagnosed with SLE during follow-up after diagnosis of ITP.

**Table 1:** Baseline characteristics of patients with ITP according to the development of SLE

	Total (*n* = 130)	No SLE (*n* = 120)	SLE (*n* = 10)	*P* value
**Age**, years	52.0 (34.0–61.0)	53.0 (36.0–62.0)	31.5 (24.0–49.8)	0.008
**F/U duration**, months	74.5 (38–118.5)	79.5 (41.3–119.5)	30 (15.5–105)	0.026
**Female sex**	90 (69.2)	81 (67.5)	9 (90.0)	0.14
**BMI**, kg/m^2^	23.8 ± 3.6	23.9 ± 3.7	22.4 ± 2.6	0.20
**HTN**	31 (23.8)	30 (25.0)	1 (10.0)	0.45
**DM**	14 (10.8)	13 (10.8)	1 (10.0)	1.00
**Clinical manifestations**				
Skin bleeding	71 (54.6)	62 (51.7)	9 (90.0)	0.022
Mucosa bleeding	22 (16.9)	20 (16.7)	2 (20.0)	0.68
Organ bleeding	17 (13.1)	11 (9.2)	6 (60.0)	< 0.001
**BM cellularity**, %	40 (30–50)	40 (30–45)	48 (30–51)	0.21
**BM megakaryocyte**, /HPF	2.8 (1.7–4.0)	2.8 (1.7–4.0)	4.1 (1.3–5.3)	0.30
**Laboratory findings**				
WBC, /μL	5800 (4675–7900)	5800 (4725–7875)	6300 (3750–8700)	0.83
Lymphocyte count, /μL	1664 (1353–2282)	1713 (1385–2308)	1253 (536–1803)	0.011
Hemoglobin, g/dL	12.9 (11.2–14.2)	13 (11.5–14.3)	10.9 (10.0–11.4)	0.001
Platelet count, × 10^9^/L	13 (7.0–25.3)	15 (7.3–26.8)	10 (4.5–13)	0.039
Pancytopenia	8 (6.2)	6 (5.0)	2 (20.0)	0.12
Leukopenia^a^	19 (14.6)	16 (13.3)	3 (30.0)	0.16
Neutropenia^b^	7 (5.4)	6 (5.0)	1 (10.0)	0.20
Lymphocytopenia^c^	46 (35.4)	39 (32.5)	7 (70.0)	0.033
Anemia^d^	42 (32.3)	34 (28.3)	8 (80.0)	0.002
Severe thrombocytopenia^e^	84 (64.6)	74 (61.7)	10 (100)	0.014
AST, IU/L	22 (17–27)	22 (17–28)	24 (19–31)	0.54
ALT, IU/L	19 (13–28)	19 (13–28)	19 (13–23)	0.65
Creatinine, mg/dL	0.7 (0.6–0.9)	0.7 (0.6–0.9)	0.7 (0.7–0.9)	0.85
eGFR, mL/min/1.73 m^2^	92 (78–110)	92 (78–110)	95 (72–109)	0.79
ANA positivity (≥ 1:160)	16 (12.3)	10 (8.3)	6 (60.0)	< 0.001

Values are median (interquartile range), mean ± standard deviation, or number (%)

*ITP* Immune thrombocytopenia, *SLE* Systemic lupus erythematosus, *F/U* Follow-up, *BMI* Body mass index, *HTN* Hypertension, *DM* Diabetes mellitus, *BM* Bone marrow, *HPF* High-power field, *WBC* White blood cell, *AST* Aspartate aminotransferase, *ALT* Alanine aminotransferase, *eGFR* Estimated glomerular filtration rate, *ANA* Antinuclear antibody

^a^Leukopenia = WBC count < 4000/μL

^b^Neutropenia = absolute neutrophil count < 1500/μL

^c^Lymphocytopenia = absolute lymphocyte count < 1500/μL

^d^Anemia = hemoglobin < 12 g/dL

^e^Severe thrombocytopenia = platelet count < 20 × 10^9^/L

### Comparison of clinical and laboratory characteristics according to the development of SLE

We compared the clinical and laboratory characteristics at ITP diagnosis according to the development of SLE. At the time of ITP diagnosis, patients who developed SLE were significantly younger (31.5 vs. 53.0 years, *P* = 0.008) and had a lower platelet count (10 × 10^9^/L vs. 15 × 10^9^/L, *P* = 0.039) than did those who did not develop SLE. All patients who were later diagnosed with SLE had had severe thrombocytopenia. The presence of skin bleeding, organ bleeding, lymphocytopenia, anemia, and ANA positivity (≥ 1:160) were significantly more common in the patients who later developed SLE. There were no significant differences in the cellularity and megakaryocyte counts in bone marrow biopsy between the two groups.

### Comparison of treatment response and medications according to the development of SLE

We compared the treatment response and medications within one year after ITP diagnosis according to the development of SLE (Table 2). In the total study population, there were 54 (41.5%) complete responders, 47 (36.2%) partial responders, and 29 (22.3%) no responders. There were no significant differences in the treatment response or the types of medications for ITP according to the development of SLE.

**Table 2:** Treatment response at 1 year after ITP diagnosis and medications used during treatment

	Total (*n* = 130)	No SLE (*n* = 120)	SLE (*n* = 10)	*P* value
**Treatment response**				1.00
Complete	54 (41.5)	50 (41.7)	4 (40.0)	
Partial	47 (36.2)	43 (35.8)	4 (40.0)	
No response	29 (22.3)	27 (22.5)	2 (20.0)	
**Medications**				
First-line treatments				
Corticosteroid use more than 1 month	108 (83.1)	99 (82.5)	9 (90.0)	1.00
Intravenous immune globulin	30 (23.1)	28 (23.3)	2 (20.0)	1.00
Anti-Rho(D) antibody	5 (3.8)	4 (3.3)	1 (10.0)	0.33
Second-line treatments				
Rituximab	2 (1.5)	2 (1.7)	0	1.00
Romiplostim	1 (0.8)	1 (0.8)	0	1.00
Eltrombopag	4 (3.1)	4 (3.3)	0	1.00
Splenectomy	14 (10.8)	13 (10.8)	1 (10.0)	1.00
Other treatments				
Azathioprine	11 (8.5)	9 (7.5)	2 (20.0)	0.20
Cyclosporine	2 (1.5)	2 (1.7)	0	1.00
Danazole	31 (23.8)	30 (25.0)	1 (10.0)	0.45
Oxymetholone	1 (0.8)	1 (0.8)	0	1.00
Helicobacter pylori eradication	18 (13.8)	17 (14.2)	1 (10.0)	1.00

Values are number (%)

*ITP* Immune thrombocytopenia, *SLE* Systemic lupus erythematosus

### Detailed characteristics of patients who developed SLE

The details of the 10 patients who developed SLE are shown in Table 3. Most of the patients who developed SLE were female (9/10, 90%); six patients had organ bleeding, and another six patients had ANA positivity (≥ 1:160). The median time from ITP diagnosis to the development of SLE was 2.5 years (IQR, 1.3–7.7). At the time of SLE diagnosis, the most common clinical symptom was arthritis (*n* = 8), followed by skin rash (*n* = 4) and fever (*n* = 4). Interestingly, ANA titers had increased between ITP diagnosis and SLE diagnosis in most patients, and high titers of ANA (> 1:320) were found in seven out of the nine patients who were tested.

**Table 3:** Clinical characteristics and laboratory findings in patients who developed SLE

N	At ITP diagnosis							At SLE diagnosis						
	Sex	Age	Organ bleeding	ANA	Platelet count (× 10^9^/L)	Lymphocyte count (/μL)	Hemoglobin (g/dL)	ITP duration	Clinical features	ANA	Platelet count (× 10^9^/L)	Lymphocyte count (/μL)	Hemoglobin (g/dL)	SLEDAI-2K
1	F	24	Vaginal bleeding	1:320 (S)	2	829	9.6	3 years	Arthritis, skin rash, fever, enteritis	1:1280 (H)	89	948	9.6	12
2	F	31	Vaginal bleeding	1:80 (S)	13	1289	12.8	19.4 years	Arthritis	1:1280 (H)	52	1305	11.6	9
3	F	32	-	< 1:40	9	2033	11.1	5.5 years	Arthritis, skin rash, alopecia, Raynaud phenomenon	1:1280 (H)	192	493	11	12
4	F	52	Intracranial hemorrhage	1:40 (S)	13	2222	10.3	2.6 years	Arthritis, skin rash, alopecia	1:80 (N)	68	2478	12.2	11
5	F	48	-	1:40 (N)	5	557	11.1	1 year	Fever	1:80 (N)	43	1699	12.6	6
6	F	24	-	1:680 (S)	8	1726	12.4	1.8 years	Arthritis, skin rash, fever	1:1280 (S)	449	1500	8.9	13
7	F	39	Vaginal bleeding	1:160 (S)	11	475	10	2.3 year	Serositis, ascites	1:1280 (S)	48	610	12.5	5
8	F	18	-	1:320 (H)	11	1444	9.9	1.3 year	Arthritis, proteinuria	1:320 (H)	103	854	11.7	12
9	F	25	Vaginal bleeding	1:1280 (S)	3	1218	10.9	14.1 year	Arthritis	NA	36	1673	12.4	7
10	M	58	Hematochezia	1:320 (N)	17	397	10.8	1.2 year	Arthritis, fever, serositis	1:320 (N)	205	2220	15	7

*SLE* systemic lupus erythematosus, *ITP* immune thrombocytopenia, *N* number, *ANA* antinuclear antibody, *SLEDAI-2K* SLE disease activity index 2000, *F* female, *M* male, *(S)* speckled type, *(H)* homogenous type, *(N)* nucleolar type, *NA* not available

### Clinical factors associated with SLE development in ITP patients

Logistic regression analysis was performed to identify the factors at the time of ITP diagnosis that were associated with the development of SLE during follow-up (Table 4). The results of the univariate analysis showed that young age (< 40 years), skin bleeding, organ bleeding, ANA positivity, lymphocytopenia, and anemia were significantly associated with the development of SLE. Furthermore, multivariate analysis revealed that young age (< 40 years; OR, 6.307 [95% CI, 1.114–34.908]; *P* = 0.035), organ bleeding (OR, 13.672 [95% CI, 2.437–76.689]; *P* = 0.003), and ANA positivity (≥ 1:160) (OR, 6.638 [95% CI, 1.399–31.504]; *P* = 0.017) were significantly associated with an increased risk of the development of SLE in patients with ITP.

**Table 4:** Factors associated with the development of SLE in patients with primary ITP

	Univariate			Multivariate		
	OR	95% CI	*P* value	OR	95% CI	*P* value
Young age^a^	5.444	1.332–22.250	0.018	6.307	1.114–34.908	0.035
Female	4.333	0.530–35.422	0.17			
BMI	0.873	0.717–1.070	0.20			
Skin bleeding	8.419	1.034–68.533	0.046			
Mucosa bleeding	1.250	0.247–6.330	0.79			
Organ bleeding	14.864	3.633–60.815	< 0.001	13.672	2.437–76.689	0.003
Platelet counts, × 10^9^/L	0.911	0.828–1.002	0.055			
ANA positivity (≥ 1:160)	16.500	3.984–68.341	< 0.001	6.638	1.399–31.504	0.017
Leukopenia^b^	2.786	0.653–11.892	0.17			
Neutropenia^c^	2.111	0.229–19.499	0.51			
Lymphocytopenia^d^	4.846	1.189–19.759	0.028			
Anemia^e^	10.118	2.044–50.091	0.005			

*SLE* Systemic lupus erythematosus, *ITP* Immune thrombocytopenia, *BMI* Body mass index, *ANA* Antinuclear antibody, *OR* Odds ratio, *CI* Confidence interval

^a^Young age = age < 40 years

^b^Leukopenia = white blood cell count < 4000/μL

^c^Neutropenia = absolute neutrophil count < 1500/μL

^d^Lymphocytopenia = absolute lymphocyte count < 1500/μL

^e^Anemia = hemoglobin < 12 g/dL

## Discussion

In the present study, we examined the clinical characteristics of patients with primary ITP who later developed SLE and identified the factors at the time of ITP diagnosis that were associated with the risk of SLE development. The incidence rate of SLE development in patients with primary ITP was 7.7%, and the development of SLE was significantly associated with young age (< 40 years), organ bleeding, and ANA positivity (≥ 1:160).

Thrombocytopenia of less than 100 × 10^9^/L platelets is one of the hematological criteria for the classification of SLE and is a common clinical manifestation with a prevalence of 7 to 30% in patients with SLE [3–5]. It has been reported that thrombocytopenia is associated with poor prognosis including higher mortality in SLE [4, 17]. Although the exact mechanism of immune-mediated thrombocytopenia in SLE is unknown, recent studies have shown that ITP and SLE share commonalities in terms of genes, pathways, and molecular signatures [6–9]. In a recent study based on the National Database in Taiwan, SLE occurred in 4.7% of patients with idiopathic ITP, and the risk of developing SLE was 26 times higher than that in non-ITP patients [12]. Interestingly, the observed crude rate of SLE development in our study (10/130, 7.7%) is similar to that reported in the previous population-based study on patients with ITP. In addition, our present study provides information on the prognostic factors for the development of SLE in patients with primary ITP.

The incidence of ITP showed a bimodal pattern according to age, with peaks among ages under 5 and over 60 years [18]. ITP has a male predominance in pediatric patients and older patients and a female predominance in reproductive age (18–49 years) populations [18]. Previous studies have shown that younger ITP patients below 40 years of age had different clinical features, including a better response to rituximab treatment than those over 40 years [19–21]. On the other hand, SLE is a typical disease that affects women of childbearing age [22]. Interestingly, in our present study, young age (< 40 years) was significantly associated with the development of SLE in ITP patients.

While ANA testing is not essential for the diagnosis of ITP, it can be helpful for differentiating autoimmune diseases such as SLE. In a previous study, the rate of ANA positivity was higher in ITP patients with SLE than in those with primary ITP only [23]. In our present study, the proportion of ANA positivity was also higher in patients who developed SLE than those who did not, and ANA positivity (≥ 1:160) at the time of ITP diagnosis was a significant risk factor for the development of SLE. Notably, most patients who developed SLE showed increases in their ANA titer compared to when it was measured at ITP diagnosis, and half of these patients showed a high ANA titer of 1:1280. Thus, these findings suggested that the test for ANA may be a useful tool in the diagnosis of ITP in particular differentiating SLE; repeated measurement may be required in some instances. The results of our study were different from that of a previous study, in which ANA testing was suggested to be unnecessary for SLE screening in patients with ITP [24]. Although the exact reason for this difference is unclear, differences in the number of study patients and the duration of follow-up may be responsible.

Internal organ bleeding is one of the most serious clinical manifestations in ITP because it may potentially lead to functional impairment in major organs or a life-threatening condition [14]. In our present study, the prevalence of severe thrombocytopenia (< 20 × 10^9^/L) was significantly higher in patients who later developed SLE than those who did not, and organ bleeding was an independent risk factor for the development of SLE. However, platelet count itself was not significantly associated with the occurrence of SLE (Table 4). The bleeding tendency in SLE may be related to various factors including renal function impairment, lupus anticoagulant, and the presence of autoantibodies against coagulation factors other than thrombocytopenia [25–27]. Thus, further studies on the mechanisms and risk factors on the bleeding diathesis in SLE will be helpful for providing proper management of bleeding manifestations in SLE.

The present study had some limitations. First, this study may have been affected by a selection bias inherent to its retrospective and single-center design. Specifically, our study included patients who underwent bone marrow examination and ANA test; however, in general, bone marrow examination is not necessary for diagnosing ITP, and the ANA test is not routinely performed for all patients with ITP. Thus, selected patients may have been included in our study. Moreover, very few (*n* = 9) patients underwent the tests for additional autoantibodies (e.g., antibodies against extractable nuclear antigens) other than ANA at baseline. Second, in order to exclude patients who had SLE at the time of ITP diagnosis, only those who were diagnosed with SLE after the establishment of ITP diagnosis were included. However, it is difficult to completely rule out whether ITP was an initial clinical symptom as one of the systemic manifestations of SLE. Despite these limitations, this is a real-world study investigating the prognostic factors associated with the development of SLE after the diagnosis of ITP.

## Conclusion

In conclusion, our present study showed that young age (< 40 years), ANA positivity, and organ bleeding at the diagnosis of ITP were significantly related to the development of SLE within 1 year following ITP diagnosis. These results suggest that continued follow-up for the detection of SLE development is needed for patients with ITP, particularly those with young age, ANA positivity, or organ bleeding.

## Data Availability

The data underlying this article cannot be shared publicly for the protection of the privacy of individuals that participated in the study. The data may be shared upon reasonable request to the corresponding author.

## Abbreviations

ITP: Immune thrombocytopenia
SLE: Systemic lupus erythematosus
ANA: Antinuclear antibody
SLEDAI-2K: SLE disease activity index 2000
IQR: Interquartile range
ORs: Odds ratios
CIs: Confidence intervals
